# Editorial: Metal and Semiconductor Nanocrystals

**DOI:** 10.3389/fchem.2019.00310

**Published:** 2019-05-07

**Authors:** Jing Zhao, Ou Chen, Jie He, Shengli Zou

**Affiliations:** ^1^Department of Chemistry and Institute of Materials Science, University of Connecticut, Storrs, CT, United States; ^2^Department of Chemistry, Brown University, Providence, RI, United States; ^3^Department of Chemistry, University of Central Florida, Orlando, FL, United States

**Keywords:** nanoparticles, metal, semiconductor, alloy, oxide, perovskite

Metal and semiconductor nanocrystals have been under the spotlight for the past two decades in the rapidly developing field of nanoscience and nanotechnology. In the early days, the research of metal and semiconductor nanocrystals largely focused on Au and Cd-based materials. More recently, a broad range of compositions have been explored, including noble metal and transition metal alloys, perovskites, lead-based semiconductors, indium-based semiconductors, etc. Owing to the advancement in wet-chemical synthesis, the shape of the nanocrystals can be exquisitely controlled with high uniformity, and reproducibility. Efforts have also been devoted to developing hybrid materials that combine metal and semiconductor nanocrystals with other nano or micro materials (such as oxides and polymers) in one structure, which lead to intriguing new properties (or synergies). Currently, metal and semiconductor nanocrystals are being applied in photonics, catalysis, biological imaging, sensing and therapeutics, lighting, energy, and so on. This Research Topic collects a series of research work (both original research and review) in the field of metal and semiconductor nanocrystals. They cover a diverse range of topics, including nanomaterial synthesis, optical and electronic properties, catalytic, and biomedical applications.

Although Au nanocrystals have been extensively studied for two decades, synthesis of well-defined complex structures with high uniformity still remains challenging. Chang et al. developed a method to synthesize Au nanooctopods using a seed-mediated method. The high uniformity of the nanootopods yields a narrow plasmon band of these nanocrystals. The nanooctopods have a high photothermal conversion efficiency (>80%), making them promising photoacoustic contrast agent. In seed mediated method to grow Au nanocrystals, sometime Ag^+^ ions were added to direct the growth. In the work reported by Athukorale et al. a mechanistic study was performed to understand the adsorption of Ag^+^ ions on Au nanocrystals. UV-vis, zeta-potential, X-ray photospectroscopy along with surface-enhanced Raman spectroscopy were applied to study the Ag^+^ adsorption process *in-situ* and *ex-situ*. The results indicate the bound Ag^+^ ions remain cationic, and there were two parallel processes occurring.

Other than Au nanocrystals, three reports focus on PtNi alloy nanocrystals, and Pt, Rh, etc nanocrystals decorated on oxide nanostructures. Pt based nanocrystals are known to be good catalysts for many chemical and electrochemical reactions. Song et al. synthesized Pt-Ni alloy nanohexapods. The unique structure makes the nanohexapods good electrocatalysts for oxygen reduction reaction. Metal nanocrystals can also be decorated onto oxides to provide new functionality. In the study by Dai et al. ultrasmall Pt and Rh nanoclusters were bound to silica nanospheres. As a result, the absorption of these ultrasmall clusters were enhanced by the resonant and random scattering of light by the silica nanospheres. In another study by Demille et al. a light-mediated growth method was developed to grow a range of metal nanocrystals (Au, Ag, Cu, Pt, Pd, Tu, Ir, and Rh) from ZnO tetrapods with nanostructures. The Pd, Rh, and Ag nanocrystals decorated ZnO tetrapods show significant catalytic activity toward 4-nitrophenol reduction.

Essentially, for all nanocatalysts, one of the critical issues is their stability. Given the large surface energy of nanocatalysts, how to retain their high surface areas and defined nanostructures during the reactions has received enormous attention. The review paper by Xu et al. summarizes the recent advances in metal nanocatalysts supported on mesoporous zeolites, with an emphasis on their catalytic activity and stability for a number of organic reactions. The methodologies on the synthesis of mesoporous zeolites with pre-designed metal nanocrystals are highlighted. The coupling of metal nanocatalysts with various zeolites is discussed in the context of the catalytic synergies.

Three nice articles covering different types of semiconductor nanocrystals are collected in the topic. The work by Abeywickrama et al. describes the synthesis of colloidal cubic PbSe nanocrystals with particle size of 20–40 nm. A two-step iso-material particle growth method was proposed and nicely presented. Given their large size, the final PbSe nanocrystals possessed bulk bandgaps due to minimized quantum confinement. Interestingly, these abnormally large PbSe nanocrystals showed drastically improved film conductivity as compared to their smaller counterparts, demonstrating their potentials in optoelectronic device applications. In addition to the IV-VI semiconductor nanocrystals, Toufanian et al. reported a systematic study of III-V InP-based core/shell semiconductor nanocrystals. Two systems, i.e., InP/ZnS(e) with a type-I (or quasi-Type-II) bandgap alignment and InP/CdS(e) with a type-II bandgap structure were synthesized with fine-tuning the core size and the shell thickness. Different strategies in controlling emission energies of the particles were discovered in these two core-shell systems. This study provides valuable insights in precisely tailoring the optical properties of III-V semiconductor nanocrystals through heterostructural and/or compositional control.

Beyond conventional semiconductor nanocrystals, lead-halide perovskite quantum dots have been explosively reported and studied in recent years, and showed great potentials in a range of applications, such as solar cells, photocatalysis, light emitting devices (LEDs)/displays. In this context, however, how to synthesize high-quality perovskite quantum dots in a large scale with high production yield still remains challenging. In our collection, Zhang et al. reported a new synthesis by modifying conventional emulsion process. Gram scale production of CH_3_NH_3_PbBr_3_ quantum dots with more than 70% chemical yield has been successfully achieved. Importantly, the resultant perovskite quantum dots showed great optical properties and were used as efficient blue emitters in LEDs with a maximum luminance of 32 cd/m^2^.

Due to their unique properties, nanocrystals of different compositions have been applied in various disciplines. However, fundamental understanding of their unique properties still stands to be improved and more detailed experimental and theoretical works are still in high demand. For example, in synthesis, controlling the size and shape of nanocrystals with a single composition has been advanced significantly, but there are still plenty of rooms for improvement in controlling the size, shape, and compositions ratio of particles with multiple composition. While the applications of those synthesized particles including photonics, catalysis, sensing, energy harvesting and conversion, and bio-imaging, have been investigated extensively, developing simple method without intensive instrumentation to characterize the synthesized particles should also be pursued. Moreover, theoretical understanding of the growth mechanism of the nanocrystals and their size, shape, and composition dependent physical properties is far left behind. All these directions are worthy exploring in the near future.

## Author Contributions

All authors listed have made a substantial, direct and intellectual contribution to the work, and approved it for publication.

### Conflict of Interest Statement

The authors declare that the research was conducted in the absence of any commercial or financial relationships that could be construed as a potential conflict of interest.

